# Editorial: Psychosis in low- and middle-income countries (LMIC)

**DOI:** 10.3389/fpsyt.2024.1396581

**Published:** 2024-04-10

**Authors:** Manuela Russo, Nikolina Jovanovic

**Affiliations:** ^1^ Centre for Implementation Science, Health Service and Population Research Department, Institute of Psychiatry, Psychology & Neuroscience, King’s College London, London, United Kingdom; ^2^ Unit for Psychological Medicine, Wolfson Institute of Population Health, Queen Mary University of London, London, United Kingdom

**Keywords:** psychosis, low- and middle-income countries, negative symptoms, clozapine, patients’ profiles, treatment pathway, traditional healing for mental illness

Current knowledge about psychosis originates almost entirely from high-income countries while little data is derived from low- and middle-income countries (LMICs). This Research Topic collects five comprehensive original research studies delving into the complex landscape of psychosis spanning Southeast Europe, Asia, and Africa. Each paper contributes uniquely to our understanding of psychosis in LMICs, exploring various facets within this complex domain from negative symptoms, to psychopharmacological treatments, and traditional healing practices. These papers utilise diverse research methodologies, encompassing cross-sectional, longitudinal, quantitative, and qualitative studies.

## Disentangling heterogeneity in schizophrenia and clozapine prescribing in Southeast European countries

Two papers from the IMPULSE study, funded by European Commission during 2018-2022, focused on the adoption of latent profile analysis to explore heterogeneity in schizophrenia, and on prescribing patterns of clozapine for psychosis respectively.

Due to its heterogeneity, identifying distinct subgroups within patients with schizophrenia is important to shed light on its pathophysiology and provide tailored treatments. Uka et al. used latent profile analysis in a sample of 103 patients with schizophrenia from Kosovo to explore how patients’ profiles based on demographic and socio-economic variables impact on negative symptoms, health status, and the quality of life. Four distinctive profiles emerged and showed a profound influence on mobility in health, quality of life, and two facets of negative symptoms (i.e., motivation and pleasure domain). These findings could potentially inform person-centre approaches to enhance the well-being and quality of life for individuals suffering with schizophrenia.

The investigation into clozapine prescription in LMICs is pertinent for several reasons. Despite clozapine stands as a gold standard for treating treatment-resistant schizophrenia, offering notable clinical and economic advantages over alternative antipsychotics, is usually under-prescribed due to potentially severe adverse effects (i.e., agranulocytosis, neutropenia, and various cardiac and metabolic complications) and to inadequate knowledge about the drug, patient noncompliance, and resource limitations for blood monitoring and treatment protocols. Whilst this data is mainly derived from Western countries, clozapine usage in LMICs remains limited and inconsistently documented. In this study, Russo et al. unveils higher prescription rates in five Southeast European (SEE) countries compared to Western Europe. However, the dosages often fall below recommended therapeutic levels, implying potential off-label usage that warrants further exploration. This study offers valuable insights into psychosis treatment practices in LMICs in Southeast Europe.

## Further exploration of negative symptoms in China

Gao et al. directed their focus towards the association between negative symptom and social functioning in a large sample (n=202) of patients with schizophrenia from China. Specifically, this study looked at dimensions of negative symptoms (i.e., motivation, expression and emotion) whose specific contributions to social functioning remained unclear. The investigation revealed a noteworthy correlation between motivation and social functioning, spotlighting motivation as a potential therapeutic target crucial for enhancing patients’ social welfare. By understanding the intricate relationship between motivation and social functioning, clinicians and researchers can potentially devise tailored strategies to address this critical aspect of patient care, ultimately ameliorating the social outcomes for individuals grappling with schizophrenia in the Chinese context.

## Traditional healing practices and practitioners in Asia and Africa

Two papers explored the traditional healing practices and practitioners: the first focused on the perceptions of treatment pathways for psychosis in Karachi, Pakistan; the other explored psychotic experiences among apprentice traditional health practitioners (THPs) in rural South Africa.

Employing a combination of individual interviews and focus groups involving individuals with psychosis, their caregivers, and service providers, Khan et al. found that a substantial portion of individuals suffering with psychosis engaged with both formal psychiatric avenues and traditional healing practices for care. Namely, five overarching themes emerged: perception of psychosis, seeking and receiving care experiences, evaluation and diagnostic approaches, service promotion, and the realities of living with psychosis. The findings underscored a dual reliance on traditional healing practices alongside conventional biomedical treatments. Therefore, it would be imperative to establish a dialogue between traditional and clinical healthcare systems. Moreover, the study shed light on a widespread lack of awareness among both formal and informal care providers regarding severe mental health conditions, and on the existence of multifaceted barriers hindering effective treatment (e.g., limited healthcare facilities and professionals, stigma, caregiver burdens, and the dissemination of misinformation by faith healers).

van der Zeijst et al. present a longitudinal study about the course of psychotic experiences among 48 apprentice THPs in rural South Africa. Within this cultural context hallucinations and other psychotic experiences are explained as calling of the ancestors to become THPs. Although the calling is considered as a gift, most people are distressed by it. This three-year follow-up study showed a significant reduction in distress associated with psychotic experiences and a decrease in the frequency of psychotic symptoms compared to the baseline assessment. This insightful study shed light on the trajectory of psychotic experiences, symptoms, and disorders among individuals undergoing the THP apprenticeship in rural South Africa. It highlighted the potential efficacy of a culturally sanctioned healing intervention, in reframing distressing experiences into positive and valued ones. This intervention not only reduced stigma, but also bolstered social empowerment and identity construction among apprentice THPs. This study’s revelations underscore the potential impact of cultural and spiritual practices, emphasizing their role in fostering positive outcomes amidst psychotic experiences. By acknowledging the significance of culturally sanctioned interventions, this study paves the way for a deeper understanding of the beneficial impact of cultural and spiritual elements in mitigating psychosis-related distress and fostering positive trajectories within this context.

## Conclusion

Socio-cultural context deeply shapes the way psychosis is conceptualised by patients and healthcare professionals, and the approaches taken to treat the illness in LMICs. Bridging traditional and formal treatment pathways is essential to improve reach and therefore life of people suffering with psychosis. Further investigation towards person-centred approaches and alignment with existing international clinical guidelines should be sought by researchers and healthcare professionals. We hope that this Research Topic draws attention on the importance of supporting research in LMICs.

## Author contributions

MR: Writing – review & editing, Writing – original draft. NJ: Writing – review & editing, Writing – original draft.

